# Development of Fabric-Based Chemical Gas Sensors for Use as Wearable Electronic Noses

**DOI:** 10.3390/s150101885

**Published:** 2015-01-16

**Authors:** Thara Seesaard, Panida Lorwongtragool, Teerakiat Kerdcharoen

**Affiliations:** 1 Materials Science and Engineering Programme, Faculty of Science, Mahidol University, Bangkok 10400, Thailand; E-Mail: thara201@hotmail.com; 2 Center of Nanoscience and Nanotechnology, Faculty of Science, Mahidol University, Bangkok 10400, Thailand; 3 Faculty of Science and Technology, Rajamangala University of Technology Suvarnabhumi, Nonthaburi 11000, Thailand; E-Mail: dang_phy@hotmail.com; 4 Department of Physics, Faculty of science, Mahidol University, Bangkok 10400, Thailand; 5 NANOTEC Center of Excellence at Mahidol University, National Nanotechnology Center, Bangkok 10400, Thailand

**Keywords:** fabric-based chemical gas sensor, embroidered sensor, electronic nose, volatile amine

## Abstract

Novel gas sensors embroidered into fabric substrates based on polymers/ SWNT-COOH nanocomposites were proposed in this paper, aiming for their use as a wearable electronic nose (e-nose). The fabric-based chemical gas sensors were fabricated by two main processes: drop coating and embroidery. Four potential polymers (PVC, cumene-PSMA, PSE and PVP)/functionalized-SWCNT sensing materials were deposited onto interdigitated electrodes previously prepared by embroidering conductive thread on a fabric substrate to make an optimal set of sensors. After preliminary trials of the obtained sensors, it was found that the sensors yielded a electrical resistance in the region of a few kilo-Ohms. The sensors were tested with various volatile compounds such as ammonium hydroxide, ethanol, pyridine, triethylamine, methanol and acetone, which are commonly found in the wastes released from the human body. These sensors were used to detect and discriminate between the body odors of different regions and exist in various forms such as the urine, armpit and exhaled breath odor. Based on a simple pattern recognition technique, we have shown that the proposed fabric-based chemical gas sensors can discriminate the human body odor from two persons.

## 1. Introduction

In the present time, sensing devices including chemical sensors have become increasingly indispensable for human wellness and security. From the point of view of the research community, it is demanding to find new sensor materials to meet new requirements from newly emerging users ranging from the military to the elderly communities. Various materials have been employed to make chemical gas sensors such as conductive polymers [1], metal oxides [2], porphyrins [3], nanocomposite materials [4,5], *etc.* These gas sensors can be individually used to detect specific gases of interest, or they can be used as an array of several sensors to collaboratively analyze complex odors. The latter is called an electronic nose (e-nose), as the technology mimics the function of the mammalian nose such as in dogs and humans. E-noses have been used in diverse industry applications such as food production [6,7], quality assessment of beverages [8,9], vegetables and fruits [10], drugs [11], chemicals [12,13], cosmetics and fragrances [14]. Interestingly, e-noses can be integrated into the production process, for examples, for grading the quality of raw materials [15], comparing a product's odor with a standard odor, checking for contaminants or impurities in a product, and imitating odors [16]. In addition, e-noses have been used in environmental monitoring such as monitoring odors of water and air pollution [17]. In the medical field, they can help in screening and diagnosis of certain diseases such as lung cancer [18] and detection of armpit odor in health care [19]. Last but not least, e-noses have been also employed in security applications, such as sniffing prohibited substances and explosive materials [20].

Most of the abovementioned gas sensors and e-nose were developed based on rigid electrodes and substrates such as wafers, glasses or ceramics. In 1996, Collins and co-workers [21] proposed a new kind of gas sensor based on fabric substrates, which was used to detect toxic gases and chemical vapors present in some types of explosives. Reynolds *et al.* have fabricated gas sensors by coating polypyrrole onto cloth fibers to measure ammonia and hydrogen chloride [22]. However, previous research on fabric-based sensors has not been focused on detecting the distinguishable volatile substances that emanate from the human body odor. In addition, work on fabric-based chemical gas sensor arrays or e-noses is also rarely found. As global demand for ubiquitous computing has soared recently, there is correspondingly increasing interest in the development of wearable devices, especially fabric-based gadgets, for health monitoring [23–25]. At present, most wearable devices are based on hybrid technology combining fabric-based sensors working under conventional non-flexible electronics with the hope of all-fabric electronics in the near future [26,27].

In the last decades, support for European projects on wearable technology for healthcare and wellness have been unprecedentedly increasing. For instance, the “Stella” project focuses on the development of stretchable electronics for large area applications on functional clothes and on integrating electronics into the stretchable parts of materials [28]. An embedded measurement system was attached to a wrist strap to detect interface pressures in chronic wound patients and to monitor their activities, to the shoe insoles to monitor diabetes and onto pajamas to monitor the respiratory system of infants. There are also other projects like “TecInTex” which aims at incorporating sensors, signal transmission and other active components into textiles. It has successfully created near infrared spectroscopy systems in socks for early detection and treatment of peripheral vascular disease (PVD) and intelligent underwear for paraplegic people to prevent ulcers [29]. In addition, the “Place It” project focuses on optoelectronic systems based on light-emitting foils, stretchable materials and textile fabrics which are very interesting for the fashion industry [30]. These research projects have yielded many useful fabrication technologies for integrating electronic functionality into textiles and clothing, as well as spun off various wearable systems for monitoring of physiological parameters and bio-kinetics such as respiration, cardiac activity and temperature of the body. However, there is no work related to on-fabric healthcare device to monitor the human body odor. Therefore, our research work will extend the functionality of wearable technology beyond the existing landscape. Moreover, the fabric-based chemical gas sensors fabricated using our processes have many advantages such as low power consumption, light weight and the ability to be plugged into various wearable platforms such as shirts and shoes. In addition, our sensors which are aimed to be consumer electronics are pervasive, affordable and easy-to-use. To our best knowledge, there exists no work on fabric-based chemical sensor arrays suitable for the development of wearable electronic nose devices. Thus, the emergence of wearable e-nose will open new frontiers in point-of-care medical diagnostics, where the human body odor can be used to indicate the health status [31]. In this study, we have therefore explored this territory by designing and fabricating amine sensors on fabric substrates and demonstrated their potential to be a wearable electronic nose. The amine sensors were made by drop coating four types of carboxylic-functionalized single-walled carbon nanotube (SWNT-COOH)/polymer solutions onto interdigitated electrodes as prepared by embroidery of conductive thread into the fabrics. Thus, the resulting sensor array can subsequently be embedded into a garment by conventional textile techniques, including the integration of the device with other electronic components. Although the present work is limited to the preparation of the sensor array and not the construction of a readily available wearable system, future construction of a wearable prototype should be conveniently possible using the design and architecture provided herein. Rather, we have focused on the validity of the sensor array as an electronic nose by demonstrating its capability to detect various vapors as related to the human body, especially amines. Based on a simple pattern recognition technique, we have shown that the proposed fabric-based chemical gas sensors can discriminate the human body odor from two persons.

## 2. Experimental Section

### 2.1. Chemicals and Materials

**Figure 1.**
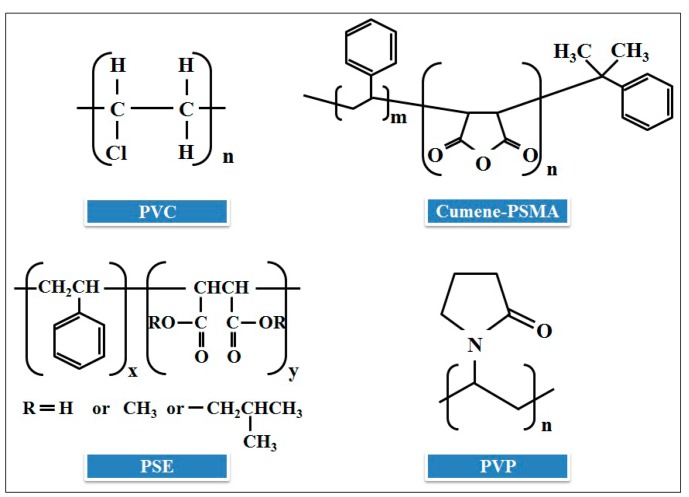
Structures of polyvinyl chloride (PVC), cumene terminated polystyrene-co-maleic anhydride (cumene-PSMA), poly(styrene-co-maleic acid) partial isobutyl/methyl mixed ester (PSE) and polyvinylpyrrolidone (PVP).

SWNT-COOH carboxylic acid functionalized single-walled carbon nanotubes with 90 wt% purity, 0.5–2.0 μm in length and 1–2 nm in diameter was supplied by Cheap Tube Inc. (Windham, VT, USA; www.cheaptubesinc.com). Four different types of polymers, namely, polyvinyl chloride (PVC), cumene terminated polystyrene-co-maleic anhydride (cumene-PSMA), poly(styrene-co-maleic acid) partial isobutyl/methyl mixed ester (PSE), and polyvinylpyrrolidone (PVP), whose structures are shown in Figure 1, were purchased from Sigma-Aldrich (St. Louis, MO, USA) and selected as matrix materials for the preparation of the conductive composite gas sensors. The mixture of each polymer with SWNT-COOH was prepared by dissolving polymer (3 mg) in a proper solvent (1 mL of tetrahydrofuran, acetone, acetone and water, respectively). Then, SWNT-COOH was blended into each polymer solution in order to obtain a high conductivity with a percent loading for SWNT-COOH:polymer of 30:70. This mixture was stirred for 30 min, followed by 30 min of continuous ultrasonic vibration at 25 °C. This process was repeated three times to ensure sample uniformity.

Cotton satin fabrics were purchased from the Electric Quilt Company (Wood, OH, USA). The fabric has 283 thread counts, 100% combed cotton satin weaved fabric. This cotton satin fabric was used as substrate for fabricating the gas sensor array and was preliminarily cleaned by acetone in order to remove impurities before being embroidered with conductive thread to construct the interdigitate pattern. The conductive thread used in this work is the silver-type thread purchased from SparkFun Electronics (Niwot, CO, USA). The silver-plated nylon 234/34 4-ply conductive thread, with a nominal diameter of 0.2 mm and linear resistance about 0.4 Ω/cm. The thread was embroidered into the fabric substrate for fabricating interdigitated electrodes.

### 2.2. Fabrication of Fabric-Based Chemical Gas Sensors

**Figure 2.**
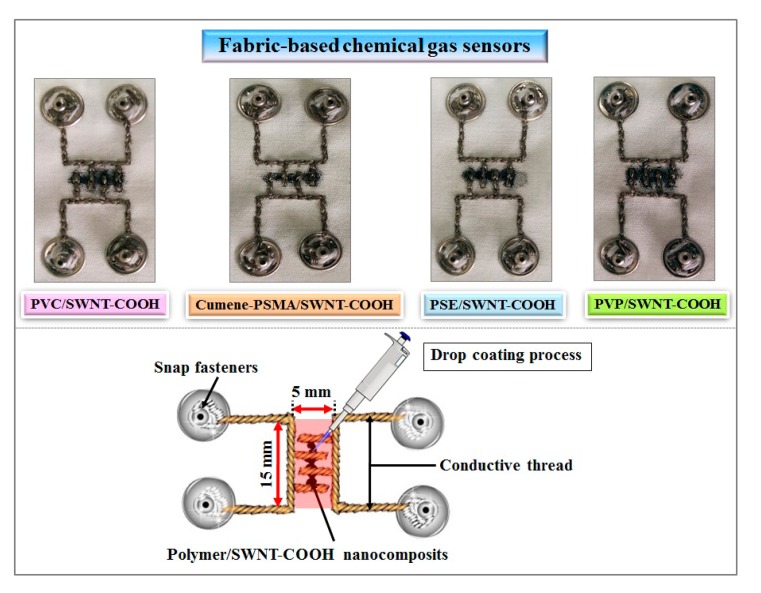
Pictures (**top**) and diagram (**bottom**) of the fabric-based chemical gas sensors.

In this work, fabrication of the fabric-based chemical gas sensors can be divided into two steps: (1) embroidery of the interdigitate electrodes and (2) drop coating the sensing materials over them. In the first step, conductive thread was used as the interdigitated electrodes based on embroidery on the fabric substrates along the lines to produce comb-like patterns as shown in Figure 2. The embroidered interdigitated electrodes were an important component of the wearable sensor to function as a sensing area, whose surface area is approximately 0.5 × 1.5 cm^2^. Conductive thread and snap fasteners were used to connect the chemical gas sensors with the external circuit port, in which external devices can be plugged into the active area to fulfill the measurement. In this paper, we have connected the sensor array to a data acquisition system, which can be further designed to be embedded into the fabrics in the future.

In the next step, the polymer/SWNT-COOH solution mixtures, as prepared by the process described in Section 2.1., were drop-coated onto the embroidered interdigitated electrodes. The electrical resistance of chemical gas sensor was measured during the drop-coating process. It was found that the electrical resistance of each sensor was approximately 1–20 kΩ. Then, the fabricated sensors were baked in an oven at a controlled the temperature of 100 °C for 1 h in order to eliminate any remaining solvents.

### 2.3. Odor Sample Preparation

To demonstrate the working of the prepared fabric-based chemical gas sensors for use as a wearable e-nose, we have employed two kinds of odor samples, namely: (1) selected volatile substances; and (2) human body odor samples from a specific part of the body, which will be examined here. The gas sensing properties of the fabric-based chemical gas sensors were investigated based on two experimental setups as follows:

(1) Static measurements were designed to investigate the sensitivity of the fabric-based chemical gas sensors (response versus vapor concentration). In this experiment, the volatiles (ammonium hydroxide, ethanol, pyridine, triethylamine, methanol and acetone) were prepared by injecting the liquids into the measurement chamber, following by evaporation of the liquids throughout. Details of the static measurement will be described in Section 2.4.1.
(2) Dynamic flow measuremente were used for realizing the real-world application of the proposed fabric-based chemical gas sensors. In the present work, we have investigated the capability of the gas sensors to determine odors emanating from the human body. Details of the dynamic flow measurement will be described in Section 2.4.2.

**Figure 3.**
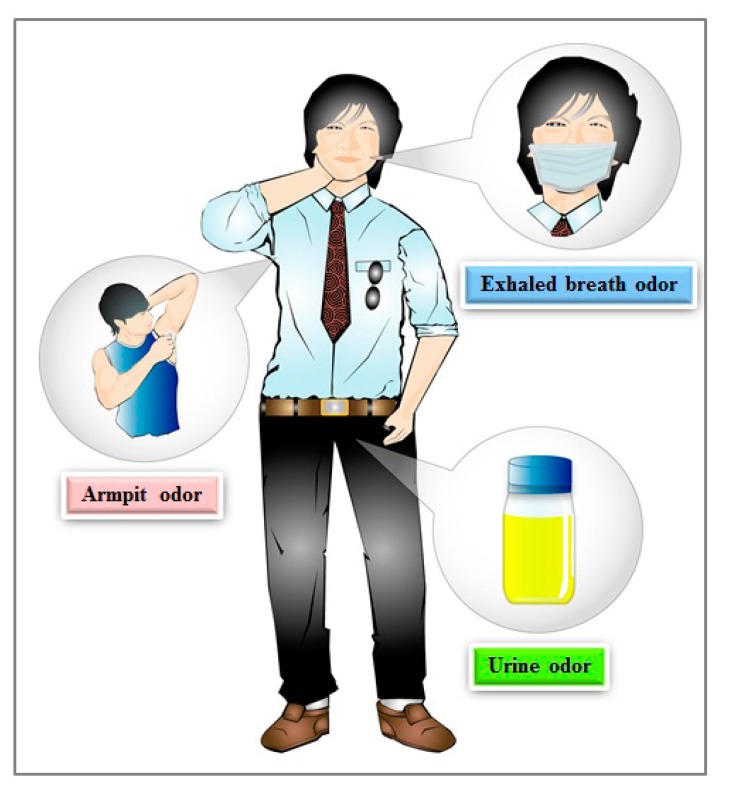
Body odor collection from volunteers for measurement.

The human body releases chemical odors via various channels. This paper explores the uses of fabric-based chemical gas sensors to monitor the human body odor [32] as released from armpit [33,34], exhaled breath [35] and urine [36], as shown in Figure 3. Unusual human body odors can indicate irregularities of the chemical system inside the body as caused by the diseases such as nephropathy, diabetes mellitus, liver cirrhosis and even liver cancer [37].

To examine the performance of our fabric-based chemical gas sensors on human odor, two male volunteers (20–30 years old) were selected to provide the odor samples. During the experiment, volunteers were asked to live a normal life and perform normal activities. For example, they were allowed to take a bath twice a day (after waking up in the morning and before going to bed) to limit the factors that might cause fluctuations in the odor samples. The volunteers were not allowed to have sex or consume any alcoholic drinks.

Body odor from the armpit of each volunteer was collected 1 hour after lunch (approx. 1:00 pm) by attaching cotton wool to the volunteer's underarms for 20 min and the sample was stored in a tight lid glass bottle, kept under a heated shield. The odor samples were measured within 30–60 min after collection at room temperature (25 °C) in order to reduce the chance of odor changes caused by bacteria.

Exhaled breath odor of the volunteers was collected by having each of them wear a mask for 15 min; after that the breath sample from the mask was analyzed to find the difference of the odor. The breath sample was collected in the morning at least 1 h after breakfast and brushing teeth, and was stored in a tightly closed glass bottle to prevent ageing.

The urine odor sample was collected as soon as the volunteers woke up in the morning before having any food or drink since the urine was then the most concentrated. Urine (20 mL) was collected during the middle period of excretion measured within 3 h after that. These conditions were set up in order to prevent the change of the odor due to the evaporation of odor molecules and the growth of bacteria.

### 2.4. Gas Sensing Measurement

#### 2.4.1. Static Measurement

**Figure 4.**
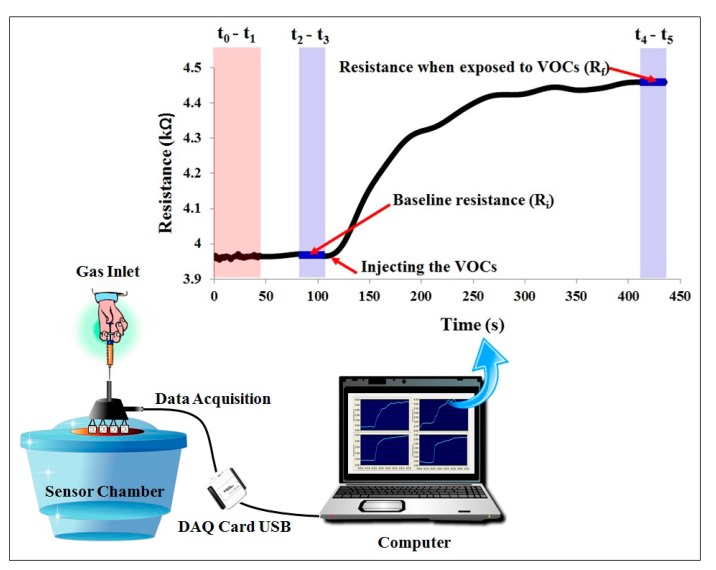
Static measurement system for detecting volatile organic compounds.

The static measurement system was set up for sensor testing, which is very important to determine the sensitivity and selectivity of each sensor to specific gases. A reasonable set of gases, as related to the planned future applications of the sensor, should be selected, which in this case are ammonium hydroxide, ethanol, pyridine, triethylamine, methanol and acetone. The static measurement system consists of three major components, namely a big gas chamber, a sensing system and a data acquisition. Four types of fabric-based chemical gas sensors were installed beneath the cover of the sensor chamber with a volume of 2000 mL. Once a liquid sample was injected onto a sample cup inside the chamber, the hot plate underneath it will help the liquid evaporate completely. In addition, a small fan has been installed inside the chamber to spread the sample vapor evenly across the chamber, as shown in Figure 4. The sensing response, as measured in term of percentage change of the electrical resistance, was measured at four gaseous concentrations (50, 200, 500 and 1000 ppm). When the experimental period ended, nitrogen gas was used to clean the chamber and sensors to prepare for the next experiment. Right before we performed a static measurement by injecting volatile organic compounds (VOCs) into the chamber, the air that was contained in the chamber would be flushed out by the nitrogen gas. This process can be demonstrated by considering a sampled signal curve as shown in Figure 4. The nitrogen was started to flush the chamber during the time t_1_-t_2_, then we would wait to stabilized the baseline before injecting the sample at the time t_3_. Then, the smooth constant signal curve during the period t_2_-t_3_ was used to represent the baseline resistance of the sensor in clean air (R_i_). After the VOC was injected at the time t_3_, we can observe an abrupt change in the sensor resistance until it stabilized at the time t_4_-t_5_ in which the signal during this period represents the sensor resistance when exposed to the tested gas vapor (R_f_).

In each experiment, the resistance of each sensor was recorded in the first 2 min to obtain background resistance, followed by a measurement for another 5 min after injection of the analyte samples into the chamber. The resistances of four fabric-based chemical gas sensors were monitored concurrently using a data acquisition card (NI USB-6008 of National Instruments, Singapore, Singapore) by LabVIEW software installed on a personal computer. The signal data was displayed in real-time on the computer screen.

#### 2.4.2. Dynamic Flow Measurement

**Figure 5.**
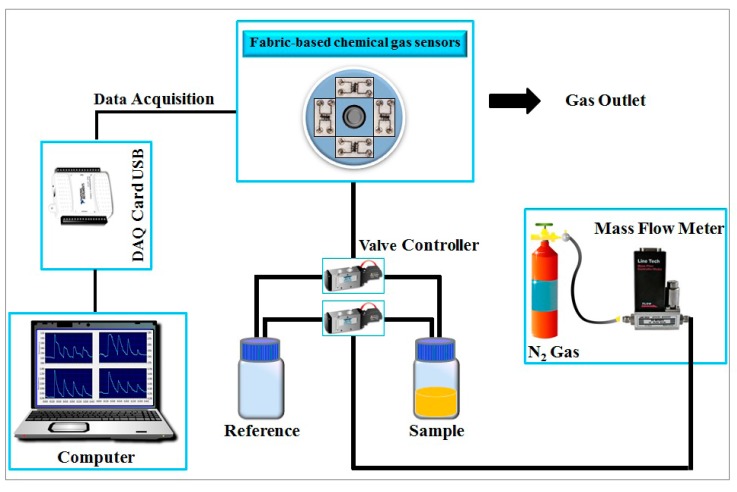
Schematic diagram of the fabric-based e-nose system.

Figure 5 shows the schematic diagram for an odor-sensing test of the fabric-based chemical gas sensors aimed to be a wearable e-nose. Note that the present design is only a preliminary version, which still does not have embedded computing functions to acquire and analyze data on the go. In order to test the functionality of the chemical sensing of this e-nose version, a dynamic flow measurement was introduced. The setup consists of three components, namely, a sample delivery system, a detection system and a computing system. We hope that this setup will simulate the situation where embedded computing function will be integrated into the wearable e-nose in the next version.

In the sample delivery system, solenoid valves are used to control the air flow by switching between the sample and reference gases. Nitrogen gas was used as the reference and to carry the sample odors to the fabric-based chemical gas sensors. A mass flow controller was used to control the flow rate of nitrogen gas at a constant rate of 0.7 L/min. The detection system contains the fabric-based chemical gas sensor array. In a practical application, this sensor array can be integrated into the surface of a shirt/garment by embroidery. In our design, snap fasteners were used to connect an external circuit to the fabric-based chemical gas sensors.

The computing system process the data acquired from the sensors using LabVIEW software, a special program used to control a USB-DAQ card, connect the devices to the computer and convert the signal from analog to digital. Each channel of the analog multiplexer selects one of several input signals from individual sensors, and forwards the selected input into a single line and sent to the output voltage signal of the four sensors.

**Figure 6.**
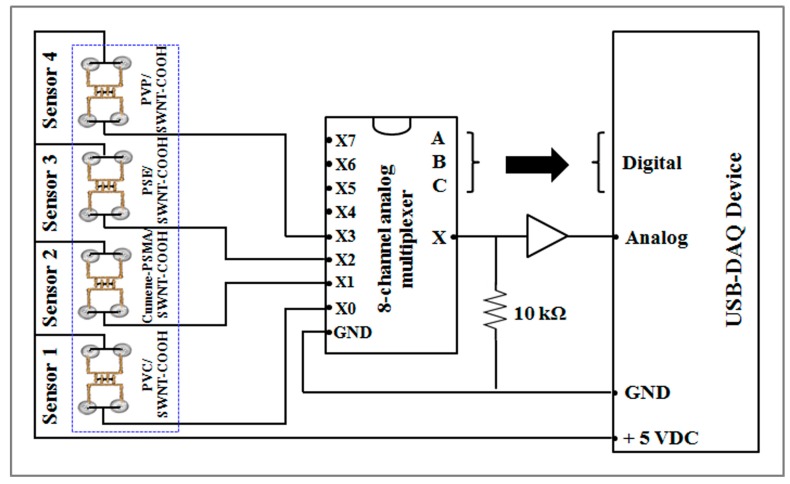
Schematic circuit diagram of data acquisition for the fabric-based e-nose.

A design of the data acquisition was presented in Figure 6. The direct current voltage at 5 V flows into the sensor circuit in order to maintain the sensor operating power.

The fabric-based chemical gas sensors have resistances between 1 kΩ to 20 kΩ. The output voltage was proportional to the equivalent resistance of the sensor. The USB-DAQ device converts the analog voltage into a digital signal for further processing by the computer. The input data from the sensor array was then analyzed based on the principal component analysis (PCA), a simple and effective method to recognize patterns in the data by visualizing them into 2-dimensional or 3-dimensional plots

## 3. Results and Discussion

### 3.1. Scanning Electron Microscopy (SEM)

**Figure 7.**
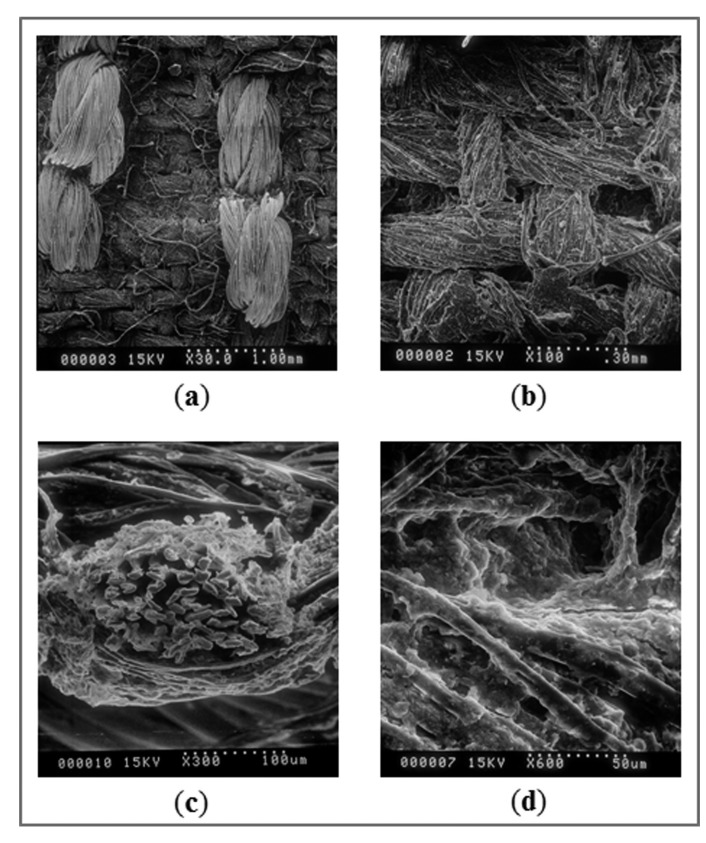
SEM pictures of (**a**) the surface of conductive thread (functioning as interdigitate electrodes) embroidered on the cotton satin fabric substrate at a magnification of 30×; (**b**) the surface of cotton satin fabric substrate at a magnification of 100×; (**c**) the cross-section of the conductive thread and the cotton satin fabrics as coated by a thick film of the polymer/SWNT-COOH nanocomposites materials 300×; and (**d**) the cross-section at 600× magnification.

Scanning electron microscopy (SEM) was performed to investigate the microstructure of the fabric-based chemical gas sensors. Figure 7a,b show the surface of the conductive thread (functioning as interdigitated electrodes) embroidered on the cotton satin fabric substrate at a magnification of 30× and 100×, respectively.

In Figure 7c,d, the cross-section of the conductive thread and the cotton satin fabrics as coated by a thick film of the polymer/SWNT-COOH nanocomposite materials was displayed at 300× and 600× magnification. It can be seen that the nanocomposite materials have penetrated into the fabrics and coated around individual fibers within the thread throughout the full thickness of the cotton satin fabrics. The rough and porous nature of the fabric surface helps to increase the percolation of the analyte gases into the sensing materials, thereby enhancing the sensing response to specific gases.

### 3.2. Sensitivity to Volatile Substances

Electrical resistances of the fabric-based chemical gas sensors were recorded before and during exposure to gas vapor. Thus, different polymers used as sensing materials have presented noticeable variations in their resistance values. The sensor response (R_r_) can be defined as percentage change in the resistance as follows:

(1) $${\text{R}}_{\text{r}}\left(\%\right)=\left[({\text{R}}_{\text{f}}-{\text{R}}_{\text{i}})/{\text{R}}_{\text{i}}\right]\times 100=(\Delta \text{R}/{\text{R}}_{\text{i}})\times 100$$

where R_i_ is the initial resistance of each sensor without the sample vapor (baseline resistance), and R_f_ is the resistances when exposed to the testing gas vapor [38].

**Figure 8.**
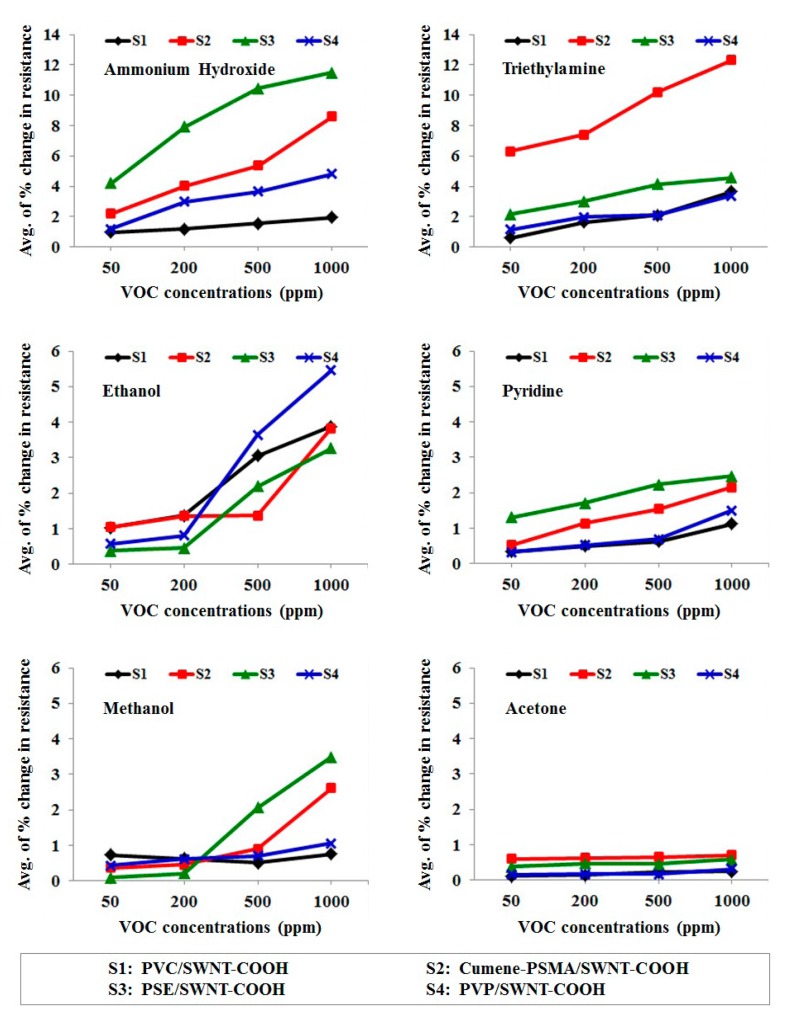
The average of percent change in resistance of fabric-based chemical gas sensors in the static measurement system when exposed to ammonium hydroxide, triethylamine, ethanol, pyridine, methanol and acetone at the concentrations of 50–1000 ppm.

Figure 8 show the average percentage change in the resistance of four fabric-based chemical gas sensors under the testing vapors, namely, ammonium hydroxide, ethanol, pyridine, triethylamine, methanol and acetone at the concentrations of 50–1000 ppm. It was found that the sensor response is rising with increasing gas concentration. According to the results, it was shown that cumene-PSMA/ SWNT-COOH and PSE/SWNT-COOH fabric-based chemical gas sensors yield the highest response to triethylamine and ammonium hydroxide, respectively. Thus, PSE/SWNT-COOH sensor presents the highest response to ammonium hydroxide, while cumene-PSMA/SWNT-COOH sensor yields the highest response to triethylamine. Other sensors such as PVC/SWNT-COOH and PVP/SWNT-COOH show much lower responses to all tested vapors. Based on structural characterization of the sensing materials, it was found that the structure of each polymer composite is quite porous. In principle, the sensing mechanism of such sensors could be explained in terms of the percolation of gas molecules into the porous film. Odorant molecules can percolate both into the microporous structure of the polymer matrix which is coated on the fiber's surface and within the fine structure of the cotton satin fabrics used as substrate. When the odorant molecules penetrate into the sensing materials on the cotton surface, the sensors' resistance increases. Swelling of the gas molecules by the polymer matrix leads to changes in the conducting channel of the carbon nanotube network due to volumetric growth. Moreover, another sensing principle that may also cause the changes in the electronic properties of the sensors is the charge transfer between odorant molecules and carbon nanotubes [39].

### 3.3. Discrimination of Odors Based on Principal Component Analysis (PCA)

Principal components analysis (PCA) is a multivariate statistical method able to reduce the dimensionality of data using a feature extraction technique based on the analysis of the covariance between the factors [40]. PCA is suitable for the multi-dimensional datasets of this work where data are collected from an array of sensors and multiple samples.

**Figure 9.**
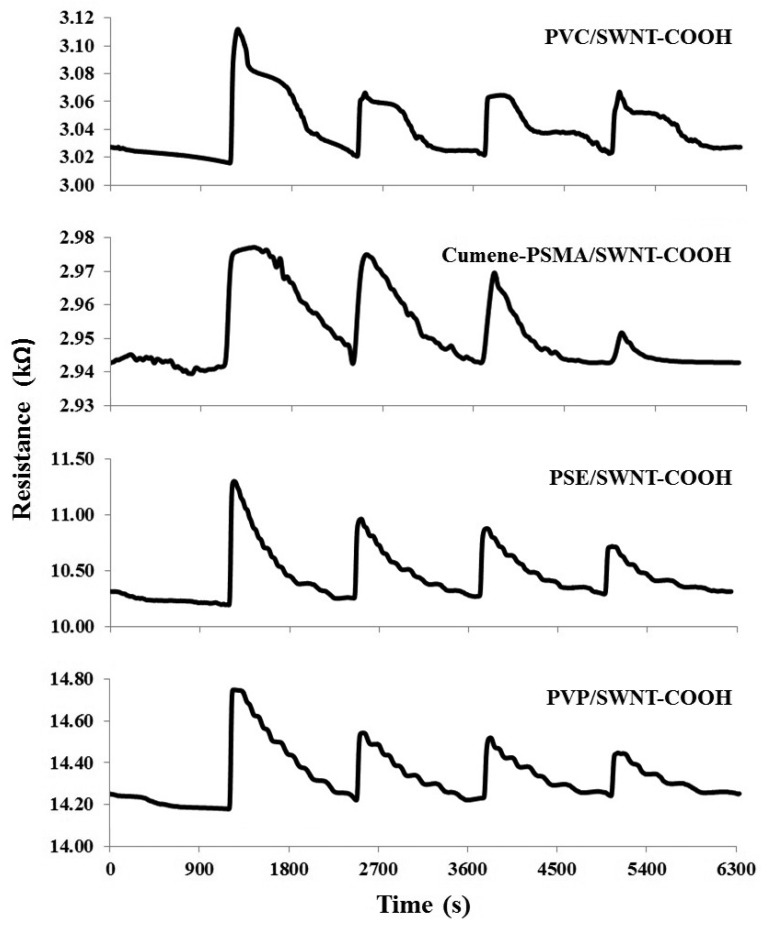
The dynamic response to urine odor by four fabric-based chemical gas sensors.

In this work, the dynamic responses of the fabric-based chemical gas sensors were recorded as a change in the resistance values. The measurement was performed by switching between the sample odor for 1 min and the purity air for 20 min. This process was repeated for four cycles. The graph of the fabric-based chemical gas sensing signal (sensogram) from four sensors is shown in Figure 9. The difference between the maximum peaks (signal from the sample odor) and the base line (signal from the reference gas) was used as feature in this analysis [41–43].

**Figure 10.**
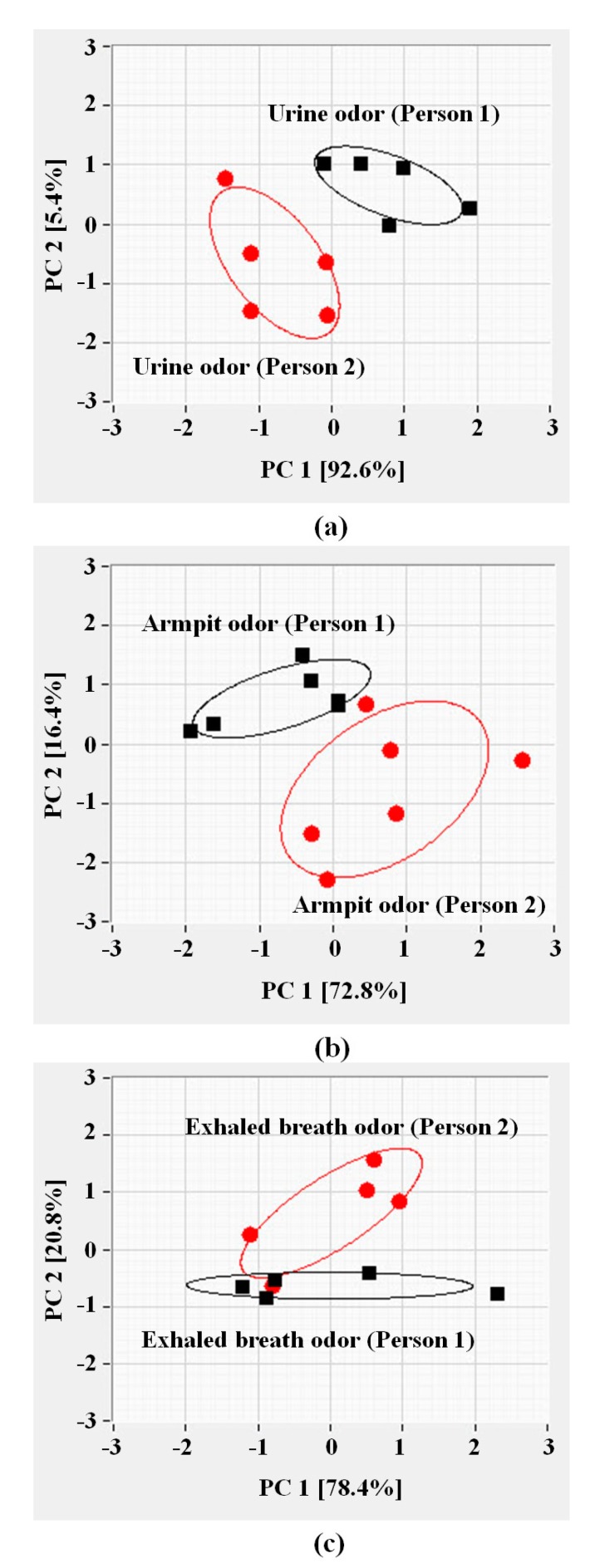
Principal component analysis (PCA) plot of the sensor data obtained from: (**a**) the urine odor; (**b**) the armpit odor; and (**c**) the exhaled breath odor of two male samples.

Figure 10a–c show the PCA results in two dimensional plots (PC1-PC2). It was found that the urine odor, armpit odor and exhaled breath odor of two male volunteers can be distinguished and grouped using the eclipses within 95% confidence. From Figure 10a, PC1 and PC2 account for 92.6% and 5.4% of the variance, respectively, which completely separate the urine odor data of person 1 from the other volunteer.

Based on PCA discrimination, it appears that urine odor can be more obviously discriminated than the armpit and exhaled breath data, respectively, in accordance with the fact that the urine odor has higher concentration of odor molecules than the odors from other parts of the body. Figure 10b shows the distinct clusters of the armpit odors from two persons which are clearly separated into two groups. PC1 accounts for the greatest variance (72.8%). Thus, it was found that the two data clusters are clearly separated on the PC2 axis, while the data points of the armpit odors are scattered along the PC1 axis.

Figure 10c shows that the exhaled breath odor of two persons can be separated into two groups on PCA plots. PC1 and PC2 contribute 78.4% and 20.8% of the total variance, respectively. The exhaled breath odors between two persons appear close together on the PCA. As a matter of fact, exhaled breath odor is less concentrated than other body odor samples. The exhaled breath odor also ages fast, leading to a decrease in odor concentration and a weak sensor response. These findings will pave the way for further development of specifically designed wearable e-nose for human body odor which is very sensitive to different odors of each person.

## 4. Conclusions

In this paper, a prototype of a fabric-based e-nose based on a polymer/SWNT-COOH nanocomposite gas sensor array was proposed for applications on human body odor monitoring. The sensors were tested with various volatile compounds commonly found in the human body such as ammonium hydroxide, ethanol, pyridine, triethylamine, methanol and acetone. These sensors were also examined with the body odors obtained from different regions and forms such as urine odor, armpit odor and exhaled breath odor. The results showed the feasibility of using this e-nose for discrimination of human body odors to indicate health status. The capability to detect various vapors as related to the human body, especially amines, has been demonstrated. Based on a simple pattern recognition technique, we have shown that the proposed fabric-based chemical gas sensors can discriminate between the human body odors from two persons. Although the present work is limited to the preparation of the sensor array and not the construction of a readily available wearable system, future construction of a wearable prototype should be conveniently done using the design and architecture provided herein.
